# AIM2 Mediates Inflammation-Associated Renal Damage in Hepatitis B Virus-Associated Glomerulonephritis by Regulating Caspase-1, IL-1**β**, and IL-18

**DOI:** 10.1155/2014/190860

**Published:** 2014-02-20

**Authors:** Junhui Zhen, Le Zhang, Jiachao Pan, Shumin Ma, Xiaojian Yu, Xiaobo Li, Shijun Chen, Wenjun Du

**Affiliations:** ^1^School of Medicine, Shandong University, Jinan 250012, China; ^2^Department of Pathology, School of Medicine, Shandong University, Jinan 250012, China; ^3^Department of Liver Disease, Jinan Infectious Disease Hospital, School of Medicine, Shandong University, Jinan 250021, China; ^4^Department of Pathology, Harbin Medical University, Harbin 150086, China; ^5^Digestive Department, Shandong Provincial Qianfoshan Hospital, Shandong University, Jingshi Road No. 16766, Jinan 250014, China

## Abstract

*Background & Aims*. AIM2 plays an important role in innate immunity, but its role in regulating the immune response to hepatitis B virus (HBV) is unknown. We hypothesized that AIM2 expression is positively correlated with HBV-mediated inflammation in patients with HBV-associated glomerulonephritis (HBV-GN), potentiating inflammation and leading to renal damage. We therefore analyzed the expression of AIM2 and inflammatory factors in HBV-GN tissues and cell lines relative to the inflammatory response to HBV infection and HBV status. *Methods*. Seventy-nine patients with chronic nephritis (CN) were included: 54 with HBV-GN and 24 with chronic glomerulonephritis (CGN). Expression of AIM2, caspase-1, and IL-1**β** was detected by immunohistochemistry in renal biopsies from each patient. Following siRNA-mediated knockdown of AIM2 in HBV-infected and HBV-uninfected human glomerular mesangial (HGM) cells, expression of caspase-1, IL-1**β**, and IL-18 was detected by qRT-PCR and Western blot. *Results*. AIM2 expression in HBV-GN biopsies (81.4%) was significantly higher than in CGN (4.0%) and positively correlated with caspase-1 and IL-1**β** expression in HBV-GN. In vitro, AIM2 knockdown reduced caspase-1, IL-1**β**, and IL-18 expression in HBV-infected and HBV-uninfected HGM cells. *Conclusion*. AIM2 elevation during HBV infection or replication may contribute to inflammatory damage, thus providing a putative therapeutic target for HBV-GN.

## 1. Introduction

Hepatitis B virus (HBV) infection is an important public health problem worldwide, especially in developing countries, and hepatitis B virus-associated glomerulonephritis (HBV-GN) remains one of the most common secondary glomerular diseases [1]. Ever since the association between HBV infection and glomerular diseases was first reported by Combes et al. in 1971 [2], more HBV-GN cases have been described all over the world. The existence of HBV DNA in the renal tissue of some nephritic syndrome patients led to the classification of HBV-GN, proposing a role for HBV in its pathogenesis [3]. However, the specific pathogenesis of HBV-GN is still unclear. The widely accepted view is that persistent viral infections could lead to immune complex-mediated nephritis [4].

HBV is a noncytopathic human hepadnavirus that causes acute and chronic hepatitis and hepatocellular carcinoma [5]. It contains a circular and partially double-stranded DNA (dsDNA) genome of approximately 3.2 kb that consists of four overlapping open reading frames—the C, S, P, and X regions. A key innate immune response to infection with microbial or viral pathogens and tissue damage is the rapid activation of multiprotein complexes called inflammasomes [5]. Hornung et al. [6] showed that cytoplasmic DNA triggers the formation of the absent melanoma 2 (AIM2) inflammasome by inducing AIM2 oligomerization. The inflammasome then activates caspase-1, a cysteine protease that processes the inactive pro-interleukin-1b (pro-IL1*β*) and pro-IL18 to their respective active proinflammatory cytokines, IL-1*β* and IL-18 [7].

AIM2 belongs to a family of HIN-200 proteins that includes at least four members in humans—IFI16, MNDA, IFIX, and AIM2. HIN-200 protein family members are interferon- (IFN-) inducible proteins with a 200-amino-acid repeat at the C-terminus, which is known as the HIN domain, and an N-terminal pyrin domain (PYD). Several studies have recently identified AIM2 as a cytosolic sensor that binds dsDNA through its C-terminal HIN domain [6, 8–12]. The AIM2 N-terminal PYD motif can recruit apoptosis-associated speck-like protein containing a CARD (ASC) and thus caspase-1, which can then lead to the formation and secretion of mature IL-1*β* and IL-18, causing subsequent tissue damage [10]. Although AIM2 is known to be involved in the host defense against microbial invasion, its role in regulating the immune response to viruses, especially to HBV, has not been well understood. Given that HBV particles have been detected in the kidneys of HBV-GN patients [3], this cytoplasmic HBV DNA could potentially be recognized by AIM2, leading to caspase-1 activation via the AIM2 inflammasome and ultimately contributing to the renal damage seen in HBV-GN patients.

In this study, we compared the expression of AIM2, caspase-1, and IL-1*β* in HBV-GN and chronic glomerulonephritis (CGN) patient kidney tissues. The effects of AIM2 expression status in primary human glomerular mesangial (HGM) cells transfected with HBV DNA or vector control on caspase-1, IL-1*β*, and IL-18 expression were also investigated. Our results showed that AIM2 expression was higher in HBV-GN tissues than in CGN tissues and was correlated with renal inflammation associated with HBV-GN. Furthermore, siRNA-mediated knockdown of AIM2 decreased the expression of caspase-1, IL-1*β*, and IL-18 in vitro. Thus, AIM2 may play an important role in the development and progression of inflammation.

## 2. Materials and Methods

### 2.1. Patients

Our retrospective study was approved by the Ethics Committee of Jinan Infectious Disease Hospital. A total of 79 patients diagnosed with chronic nephritis, identified between 2008 and 2011 at Jinan Infectious Disease Hospital and Qilu Hospital of Shandong University Shandong, China, were included in the study. The experimental group consisted of 54 HBV-GN patients, and the negative control group consisted of 25 CGN patients. Subjects received kidney puncture biopsy under ultrasound guidance to obtain tissue for diagnosis and subsequent research. Participation was dependent upon fulfillment of the following criteria: (1) patients must not have used an immune agent or antiviral agent in the past three months; (2) patients must not have HAV, HCV, HDV, HEV, or HIV coinfection; (3) patients must not have a history or current evidence of secondary glomerulonephritis; and (4) consent for participation must have been obtained from those who participated [13].

### 2.2. Diagnosis of HBV-GN and CGN

The diagnostic criteria used for CGN and HBV-GN were in accordance with the 2002 Kidney Disease Outcome Quality Initiative (K/DOQI), edited by the National Kidney Foundation (NKF) [14]. The diagnosis of HBV-GN was confirmed by pathology. Frozen slices from biopsies of the 54 HBV-GN patients were kept in a low-temperature freezer. Monoclonal goat-anti-human HBsAg and HBcAg antibodies were purchased from Dako (Denmark), and immunohistochemical staining for HBsAg and HBcAg in renal biopsies was used to confirm the diagnosis. For HBV-GN patients with undetectable HBsAg or HBcAg in the kidney tissue, HBV was detected using the JCM-6000 scanning electron microscope from Jeol, Ltd. (Japan) [15].

### 2.3. Immunohistochemistry and Scoring

Tissue specimens were first fixed in 10% formalin, and then the tissue was cut, dehydrated, dipped in wax, embedded, and sectioned. These sections were then placed on slides, baked, placed into xylene, cleared of the wax, rehydrated using graded ethanol, and immersed in 0.3% hydrogen peroxide for 5 min to reduce nonspecific background staining caused by endogenous peroxidase. The slides were then washed with PBS buffer three times for five minutes each and placed in citrate buffer solution at a pH of 6.0 and then into a high temperature pressure pot to recover the tissue antigen. After being heated, the slides were cooled and restored at room temperature, washed three more times in PBS buffer, and incubated with AIM2 rabbit anti-human polyclonal antibody (ab93015; Abcam, Cambridge, UK), caspase-1 mouse anti-human polyclonal antibody (sc-56063; Santa Cruz Biotechnology Inc., Texas, USA), and IL-1*β* rabbit anti-human polyclonal antibody (ab2105; Abcam, Cambridge, UK). The slides were then placed in a 4°C refrigerator overnight. The next day, the slides were washed with PBS buffer three times, each time lasting longer than 5 min, then incubated with the secondary antibody PV-9000 (universal antibody) at 37°C for 10 min, and washed with PBS buffer, and DAB staining was applied. The stain was terminated using running water; then the slides were washed with hydrochloric acid alcohol for differentiation. Lastly, the slides were washed with distilled water, cleared with xylene, and mounted. Meanwhile, the AIM2, caspase-1, and IL-1*β* staining with PBS substituted primary antibody respectively in HBV-GN tissue, followed by DAB, was shown as negative control.

Appearance of a tan stain in the cytoplasm signaled positive expression of the protein. After staining, scores were assigned based on stain intensity and percentage of positive cells as follows: for stain intensity, a score of 0 was given for no brown staining (i.e., no cells stained), 1 for light brown, 2 for brown, and 3 for dark brown; for percentage of positive cells, a score of 0 was given for fewer than 5% positive cells, 1 for 5% to 30%, 2 for 30% to 60%, and 3 for greater than 60%. Scores for stain intensity and percent positive were then added together, and a negative sign (−) was assigned for scores totaling 0, mildly positive (+) for scores between 1 and 3, moderately positive (++) for scores between 4 and 6, and strongly positive (+++) for scores greater than 7.

### 2.4. Cell Culture

The human glomerular mesangial (HGM) cell line used in this study was purchased from ScienCell Research Laboratories (California, USA) and isolated from human renal tissue. HRMC are cryopreserved after purification and delivered frozen. Each vial contains >5 × 10^5^ cells in 1 mL volume. HRMC are characterized by immunofluorescent methods with antibodies to fibronectin, Thy-1, and smooth muscle actin. HRMC are negative for HIV-1, HBV, HCV, mycoplasma, bacteria, yeast, and fungi. Cells were cultured in DMEM (Gibco, California, USA), supplemented with 10% fetal bovine serum (Gibco, California, USA), 100 U/mL penicillin, and 100 *μ*g/mL streptomycin at 37°C in a humidified atmosphere containing 5% CO2. Medium was changed every other day.

### 2.5. Transfection Procedures

HBV expression plasmids were constructed with a pcDNA3.0 vector. The 1.1-fold overlength HBV genome was cloned into the pcDNA3 vector to generate pcDNA3.0-1.1HBVDNA, 1.1HBV as the expression gene and ampicillin resistance for antibiotic selection (Amresco, Pennsylvania, USA). An empty expression plasmid of the same type was used as a control. Plasmid sequences were analyzed by DNA sequencing.

siRNA was designed and synthesized by GenePharma (Shanghai, China). AIM2-siRNA was synthesized accordingly: sense, 5-GUC CCG CUG AAC AUU AUC ATT-3; antisense, 5-UGA UAA UGU UCA GCGGGA CTT-3. Nontargeting siRNA pool constructs were used as a negative control (SiCONTROL). Negative control siRNA was synthesized accordingly: sense, 5-UUC UCC GAA CGU GUC ACG UTT-3; antisense, 5-ACG UGA CAC GUU CGG AGA ATT-3. Transient transfection of siRNA and plasmids was performed using Lipofectamine 2000 (Invitrogen, California, USA). In brief, HGM cells were seeded the day prior to transfection at a density of 3 × 10^5^ cells/well in a 6-well plate with complete media. For each well, 5 *μ*L siRNA or plasmid (4 *μ*g) was added into 250 *μ*L Opti-MEM (Gibco, California, USA) medium, and then siRNA or plasmids were mixed with Lipofectamine 2000. The mixture was added to cells and incubated for 6 h before replacing the medium. Protein and mRNA analyses were carried out at 48 h and 24–48 h, respectively.

### 2.6. RNA Isolation and Quantitative Real-Time PCR

Total RNA was isolated using TRIzol RNA reagent (Invitrogen, California, USA). The concentration of RNA was determined by spectrophotometry at 260 nm. Quantitative real-time RT-PCR (qRT-PCR) was used to confirm the expression levels of mRNAs. Reverse transcription was performed using the RevertAid First Stand cDNA Synthesis Kit (Thermo Fisher Scientific, Massachusetts, USA), according to the manufacturer's instructions. RNA and cDNA samples were stored at −80°C. The primer pairs used to detect AIM2 and the internal control genes are listed in Table 1. qRT-PCR was performed using a Bio-Rad Cycler CFX96 detection system (Bio-Rad Laboratories, California, USA) and SYBR Premix Ex Taq (Takara Bio, Tokyo, Japan), according to the manufacturer's instructions. Briefly, 2 *μ*L cDNA template was used for each reaction in a total volume of 20 *μ*L. All reactions were performed in triplicate. The relative quantification of target gene expression was evaluated using the comparative cycle threshold (Ct) method.

### 2.7. Western Blot

Before harvest, cells in 6-well plates were washed twice in PBS and lysed in RIPA lysis buffer (Beyotime Institute of Biotechnology, Nantong, China). Lysates were centrifuged at 12,000 g at 4°C for 10 min. Protein content of the samples was determined by the Bradford assay using bovine serum albumin (BSA) as a standard. Proteins were separated by SDS, polyacrylamide gel electrophoresis in Tris/glycine buffer (25 mM Tris and 250 mM glycine), and electroblotted onto polyvinylidene fluoride (PVDF) membranes (0.45 *μ*m, Immobilon-P; Millipore, Massachusetts, USA) followed by blocking in Tris-buffered saline with Tween 20 (TBST) containing 5% w/v fat-free milk and 0.05% v/v Tween 20. Membranes were incubated with rabbit monoclonal antibody specific for human AIM2 (ab93015; Abcam, Cambridge, UK) (1 : 200 diluted in primary antibody dilution buffer), rabbit monoclonal antibody specific for human caspase-1 (3345-1; Epitomics, California, USA) (1 : 1000 diluted in primary antibody dilution buffer), rabbit monoclonal antibodies specific for human IL-1*β* (ab2105; Abcam, Cambridge, UK) (1 : 200 diluted in primary antibody dilution buffer) and IL-18 (ab137664; Abcam, Cambridge, UK) (1 : 500 diluted in primary antibody dilution buffer) overnight at 4°C, and IL-6 rabbit anti-human polyclonal antibody (ab6672; Abcam, Cambridge, UK) (1 : 500 diluted in primary antibody dilution buffer). After washing in TBST, the membranes were incubated with horseradish peroxidase- (HRP-) conjugated secondary antibody (CWBio, Beijing, China) diluted 1 : 10,000 in TBST and incubated for 1 h at room temperature. After washing extensively in TBST, membranes were immersed in ECL detection reagent (Beyotime Institute Biotechnology, Nantong, China) followed by exposure to ChemiDoc XRS + system (Bio-Rad, California, USA).

### 2.8. Statistical Analysis

The SPSS program (version 17.0) was used for analysis. Measurement data was described as mean  ± standard deviation. Background factors were compared using Student's *t*-test (numerical data) or the Chi-square test (categorical data). Spearman's two-tailed test was used for correlation analysis, and differences were regarded as significant if the *P* value was less than 0.05 on either side.

## 3. Results

### 3.1. Expression of AIM2 Was Significantly Higher in HBV-GN Tissue than in CGN Tissue

The expression of AIM2 in biopsied kidney tissue from 54 HBV-GN and 25 CGN patients was determined by immunohistochemistry. The results showed that AIM2 expression was exclusive to the cellar cytoplasm of glomerular endothelial cells and mesangial cells in the tissue. Statistical analysis revealed that the positive expression rate of AIM2 in HBV-GN patients was significantly higher than in CGN patients (81.4% versus 4.0%, *P* < 0.01) (Table 2). Notably, AIM2 expression was not affected by age (*P* = 0.06) or gender (*P* = 0.527).

To further clarify the factors affecting the expression of AIM2 in HBV-GN tissue, we considered the potential influence of age, gender, and HBeAg status in the serum. As summarized in Table 3, the results showed that AIM2 expression was not affected by age (*P* = 0.937) or gender (*P* = 0.627). Using ELISA, serum HBeAg was detected in 47 of the 54 HBV-GN patients, showing that AIM2 expression was also not affected by HBeAg status (*P* = 0.614). Lastly, as summarized in Table 4, AIM2 expression was not affected by various HBV antigen types deposited in the kidney tissue (*P* = 0.511) [13].

### 3.2. Expression of AIM2 Was Positively Correlated with Inflammation in HBV-GN Tissue

**Figure 1:**
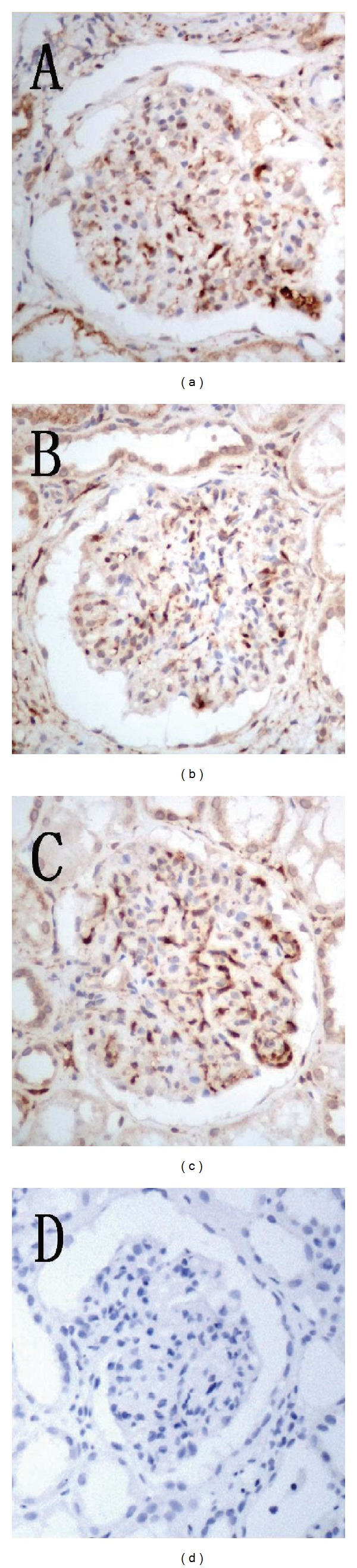
Immunohistochemical staining. (a) AIM2 positive staining in glomerular endothelial cells and mesangial cells in HBV-GN (magnification of 400x). (b) Caspase-1 positive staining in glomerular endothelial cells and mesangial cells in HBV-GN (magnification of 400x). (c) IL-1*β* positive staining in glomerular endothelial cells and mesangial cells in HBV-GN (magnification of 400x). (d) AIM2, caspase-1, and IL-1*β* negative staining with PBS substituted primary antibody in glomerular endothelial cells and mesangial cells in HBV-GN (magnification of 400x).

The correlation between the expression of AIM2, caspase-1, and IL-1*β* in patient tissues was analyzed. Statistical analysis revealed that the expression of AIM2 was positively correlated with that of caspase-1 (*r*_s_ = 0.444, *P* < 0.01) and IL-1*β* (*r*_s_ = 0.379, *P* < 0.01), and the expression of caspase-1 was positively correlated with that of IL-1*β* (*r*_s_ = 0.515, *P* < 0.01) (Table 5) [13]. Figures 1(a), 1(b), and 1(c) illustrate the positive expression of AIM2, caspase-1, and IL-1*β*, respectively, in these patients, and Figure 1(d) shows the negative expression of AIM2, caspase-1, and IL-1*β* with PBS substituted primary antibody in HBV-GN tissue. These results suggested that the expression of AIM2 was specifically correlated with inflammation in HBV-GN tissue and that elevation of AIM2 corresponding to HBV infection or replication may contribute to the inflammatory damage associated with the development of HBV-GN.

### 3.3. Expression of AIM2 Was Positively Correlated with Inflammatory Cytokine Expression in HBV-Infected HGM Cells

**Figure 2:**
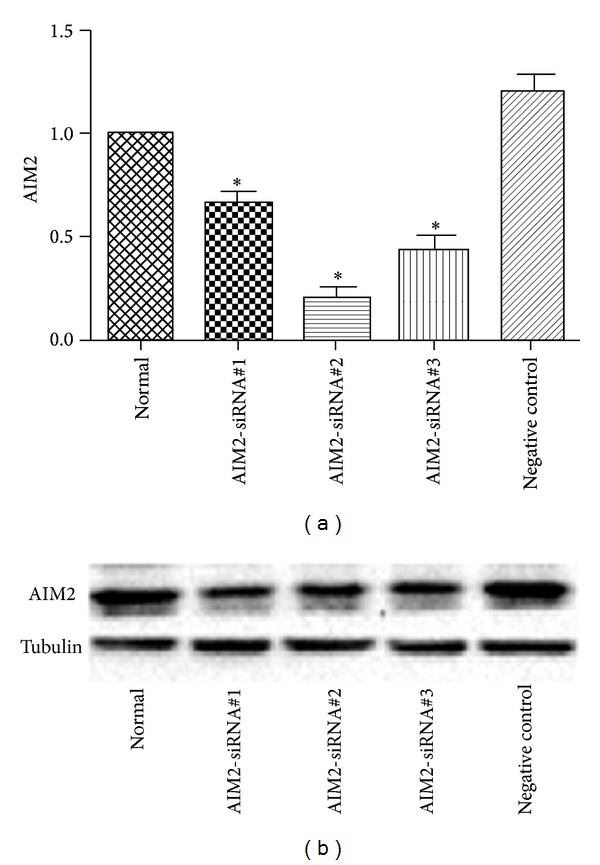
Expression of AIM2 mRNA (a) and protein (b) in transiently transfected HGM cells. AIM2-siRNA#2 was identified as the most effective silencer. AIM2-siRNA was transiently transfected to HGM cells. AIM2-siRNA#1, siRNA#2, siRNA#3 resulted in reduction of expression of AIM2 detected by qRT-PCR with 0.5, 3.8 and 1.3 folds respectively (a). These changes were also noted at the translational level, as measured by Western blot (b). *indicates a significant difference compared with the control (*P* < 0.01). *n* = 3 independent experiment RNA samples for each group.

**Figure 3:**
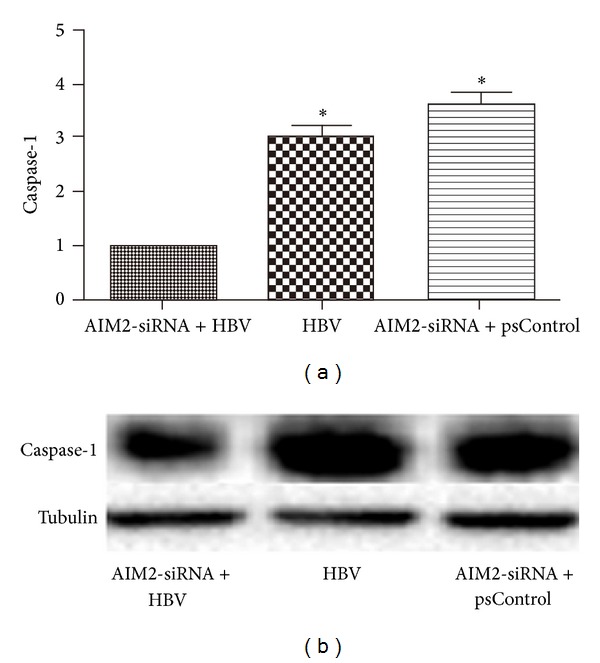
Down-regulation of AIM2 reduced expression of caspase-1 at the post-transcriptional level (a) and at the translational level (b). HGM cells were divided into three groups: AIM2-siRNA and HBV DNA co-transfected group (group A), HBV DNA-transfected group without AIM2-siRNA (group B) and AIM2-siRNA and empty plasmids co-transfected group (group C). The expression of caspase-1 in group A detected by qRT-PCR decreased in 1.9, 2.4 folds respectively compared with group B and group C (a). These changes were also noted at the translational level, as measured by Western blot (b). *indicates a significant difference compared with the control (*P* < 0.01). *n* = 3 independent experiment RNA samples for each group.

**Figure 4:**
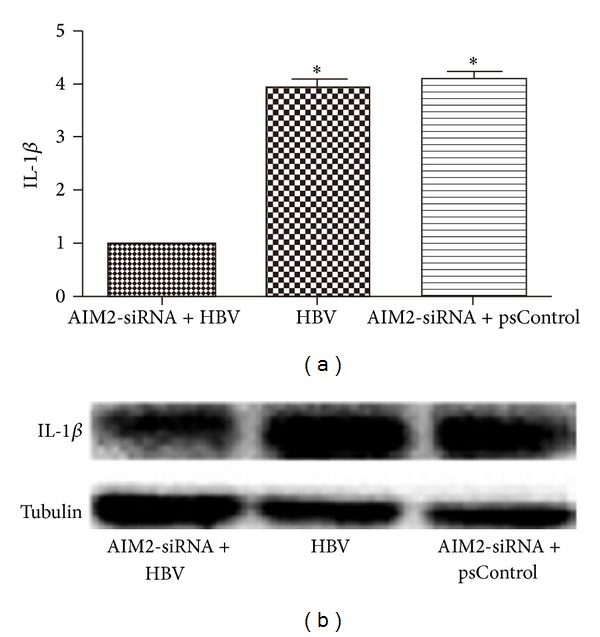
Down-regulation of AIM2 reduced expression of IL-1*β* at the post-transcriptional level (a) and at the translational level (b). HGM cells were divided into three groups: AIM2-siRNA and HBV DNA co-transfected group (group A), HBV DNA-transfected group without AIM2-siRNA (group B) and AIM2-siRNA and empty plasmids co-transfected group (group C). The expression of IL-1*β* in group A detected by qRT-PCR decreased in 2.8, 3.3 folds respectively compared with group B and group C (a). These changes were also noted at the translational level, as measured by Western blot (b). *indicates a significant difference compared with the control (*P* < 0.01). *n* = 3 independent experiment RNA samples for each group.

**Figure 5:**
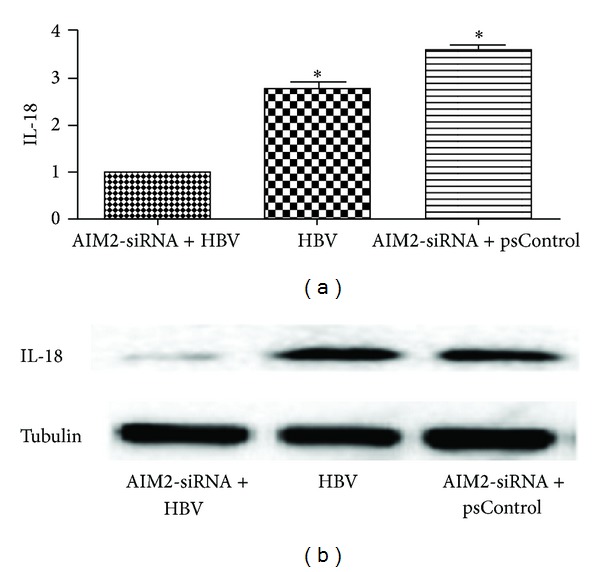
Down-regulation of AIM2 reduced expression of IL-18 at the post-transcriptional level (a) and at the translational level (b). HGM cells were divided into three groups: AIM2-siRNA and HBV DNA co-transfected group (group A), HBV DNA-transfected group without AIM2-siRNA (group B) and AIM2-siRNA and empty plasmids co-transfected group (group C). The expression of IL-18 in group A detected by qRT-PCR decreased in 1.8, 2.7 folds respectively compared with group B and group C (a). These changes were also noted at the translational level, as measured by Western blot (b). *indicates a significant difference compared with the control (*P* < 0.01). *n* = 3 independent experiment RNA samples for each group.

**Figure 6:**
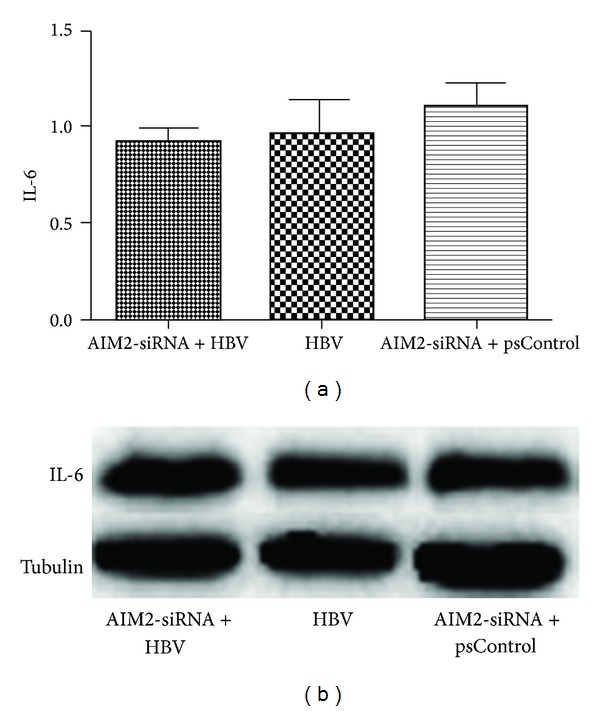
Downregulation of AIM2 had no influence on the expression of IL-6. HGM cells were divided into three groups: AIM2-siRNA and HBV DNA cotransfected group (group A), HBV DNA-transfected group without AIM2-siRNA (group B) and AIM2-siRNA, and empty plasmids cotransfected group (group C). The expression of IL-6 in group A detected by qRT-PCR had not changed compared with group B and group C (a). These similar changes were also noted at the translational level, as measured by Western blot (b). *n* = 3 independent experiment RNA samples for each group.

HGM cells were divided into three groups: AIM2-siRNA and HBV DNA cotransfected group (group A), HBV DNA-transfected group without AIM2-siRNA (group B), and AIM2-siRNA and empty plasmids cotransfected group (group C). Three AIM2-siRNAs were designed and used for this experiment. As shown in Figure 2, the expression of AIM2 mRNA and protein was tested in transiently transfected HGM cells. AIM2-siRNA#2 was identified as the most effective silencer, resulting in a 3.8-fold inhibition, and was therefore selected as a positive control for the following experiments. The expression of caspase-1 (Figure 3), IL-1*β* (Figure 4), and IL-18 (Figure 5) genes was measured at the posttranscriptional level by qRT-PCR and normalized to *β*-actin mRNA (mean ± SE, *n* = 3). Compared to group B, siRNA-mediated knockdown of AIM2 in group A resulted in a 1.9-fold decrease of caspase-1, a 2.8-fold decrease of IL-1*β*, and a 1.8-fold decrease of IL-18 mRNA levels. Compared to group C, the expression of caspase-1, IL-1*β*, and IL-18 in group A was downregulated 2.4-, 3.3-, and 2.7-fold, respectively. These changes were also noted at the translational level, as measured by Western bolt. Overall, the expression of caspase-1, IL-1*β*, and IL-18 was reduced in group A compared to the controls at both the posttranscriptional and translational levels. With AIM2 knockdown, expression of all genes studied including caspase-1, IL-1*β*, and IL-18 was decreased. In order to exclude the changes caused by AIM2-siRNA degrading all mRNA, we detected the mRNA and protein of IL-6 after siRNA transfection as control, and the results showed that the expression of IL-6 in group A detected by qRT-PCR had not changed compared with group B and group C(A). These similar changes were also noted at the translational level, as measured by Western blot (Figure 6). Taken together, these results suggested that downregulation of AIM2 could inhibit inflammation in HBV-GN.

## 4. Discussion

Increasing epidemiological, clinical, and immunological evidence has suggested a relationship between HBV infection and the development of nephropathy. However, the role of innate immunity in defense against HBV remains controversial [15]. HBV is currently viewed as a noncytopathic virus, and HBV-associated renal damage is thought to be the consequence of a long lasting cytolytic immune response against infected renal tissue. Both innate and adaptive arms of the immune system are generally involved in responding to viral infection, with innate responses being important for control of viral replication and dissemination very early after infection, as well as for timely orchestration of virus-specific adaptive responses. Activation of inflammasome complexes is a key innate immune response against infection with microbial or viral pathogens and tissue damage, and the AIM2 inflammasome appears to be an important mediator of this process in HBV-GN.

AIM2 was first reported to act as a putative tumor suppressor in malignant melanoma [16], but the AIM2 inflammasome complex is more widely known as an essential mediator of host defense against cytosolic bacteria and DNA viruses. The AIM2 inflammasome is a cytosolic nucleic acid sensor that detects microbial DNA in the cytoplasm—a compartment in which host DNA is usually not present. DNA is physiologically stored in the nucleus and in the mitochondria, and this compartmentalization of nucleic acids within subcellular organelles enables the host to distinguish self from nonself [17]. Binding of multiple AIM2 proteins to a single molecule of dsDNA could lead to a process mimicking NLR core structure formation [6]. Recent research has demonstrated that AIM2 senses potentially dangerous cytoplasmic dsDNA and forms the inflammasome with ASC, which then activates caspase-1 and the subsequent release of mature IL-1*β* and IL-18 [10], ultimately leading to tissue damage [18]. As a nonspecific receptor for cytoplasmic DNA, AIM2 can be activated by and bind to plasmid DNA, DNA from the bacterium *L. monocytogenes* and even synthetic dsDNA [11]. AIM2 has also been detected in the small intestine, spleen, peripheral white blood cells, and testis [19].

This study describes the expression of AIM2 in renal tissue and the exclusive expression of AIM2 in glomerular endothelial cell and mesangial cell cytoplasm in kidney tissue. The expression of AIM2 in the HBV-GN group was significantly higher than in the CGN group. As neither age nor gender was statistically different between the two groups, this suggests that there is indeed a relationship between chronic HBV infection and AIM2 elevation. We also considered the potential influence of HBeAg status in serum and tissue, which may have contributed to mutation of the HBV DNA-P-BCP (basal core promoter) or pre-C region, affecting activation and binding of AIM2 to HBV DNA. Importantly, the expression of AIM2 was not significantly different between the HBeAg positive and negative groups; the same results were shown for other HBV-associated antigens deposited in renal tissue groups, collectively indicating that AIM2 activation and binding to HBV DNA are not influenced by serum HBeAg status or HBV-associated antigen deposits.

IL-1*β* is an important intracellular cytokine which binds to receptors and activates the downstream NF-*κ*B signaling pathway, releasing inflammatory factors that cause tissue damage. IL-1*β* governs the recruitment of inflammatory cells such as neutrophils to the site of infection and is important in the generation of optimal adaptive immunity. IL-1*β* is not only essential to innate immune defense, but is also an important mediator of adaptive immune response to viral infections. Detailed array analyses of immediate response genes triggered by HBV identify IL-1*β* as the central activator of further adaptive immune responses [20]. In this study, expression of IL-1*β* and IL-18 from HGM cells was increased by HBV DNA. IL-1*β* and IL-18 play an important role in local immunity and in the activation of lymphocytes and macrophages in the antiviral response [21, 22], while IL-18 is mainly involved in coordinating IFN-*γ* production from NK cells and T cells at the early and late phases of infection, respectively.

In this study, the expression of AIM2 was positively correlated with the expression of caspase-1, IL-1*β*, and IL-18 in HBV-GN. Indeed, immunohistochemistry analysis showed positive staining of AIM2, caspase-1, and IL-1*β* in glomerular endothelial cells and mesangial cells in HBV-GN tissue but negative staining in CGN tissue. Within the experimental group, the expression level of AIM2 was positively correlated with the level of caspase-1, and the level of caspase-1 was positively correlated with the levels of IL-1*β* and IL-18, suggesting that the AIM2 inflammasome-caspase-1 inflammation signal transfer pathway may be active in HBV-GN. The data further suggest that downregulation of AIM2 could directly inhibit the expression of caspase-1 mRNA and subsequently inhibit the expression of IL-1*β* and IL-18 at both the transcriptional and protein levels in HBV-infected HGM cells, evident by the results of siRNA-mediated knockdown of AIM2 in HBV-infected cells. In summary, we found the expression of AIM2, a cytosolic DNA sensor, to be significantly higher in HBV-GN patient kidney tissues than in CGN tissues. Furthermore, AIM2 levels were highly correlated with inflammation in HBV-GN. The likely binding of HBV DNA to AIM2 appears to trigger activation of caspase-1 and subsequent release of IL-1*β* and IL-18, leading to renal damage. Our findings may help provide a new therapeutic target for HBV-GN and a new avenue for researching its pathogenesis and therapeutic options.

## Figures and Tables

**Table 1:** Primer sets and controls used to detect AIM2, caspase-1, IL-1*β*, and IL-18.

Oligo name	Sequence (5′-3′)	Annealing temperature (°C)	Expected length (bp)
GADPH	F: AGAAGGCTGGGGCTCATTTG R: AGGGGCCATCCACAGTCTTC	56	258
AIM2	F: TCAAGCTGAAATGAGTCCTGC R: CTTGGGTCTCAAACGTGAAGG	56	206
caspase-1	F: GCTTTCTGCTCTTCCACACC R: CATCTGGCTGCTCAAATGAA	56	160
IL-1*β*	F: GCACAAGGCACAACAGGCTGC R: CAGGTCCTGGAAGGAGCACTTCA	61	188
IL-18	F: GGAATTGTCTCCCAGTGCAT R: ACTGGTTCAGCAGCCATCTT	56	177

**Table 2:** AIM2 expression in HBV-GN and CGN.

Group	Tissue	*n*	Age	Gender M (%)	AIM2				Positive rate (%)
					−	+	++	+++	
HBV-GN	K	54	36.1 ± 12.7	35 (64.8)	10	26	18	0 0	81.4
CGN	K	25	38.2 ± 15.5*	18 (72.0)**	24	1	0		4.0***

HBV-GN, hepatitis B virus-associated glomerulonephritis; CGN: chronic glomerulonephritis; K: kidney; M: male; *compared with HBV-GN, (*t* = −1.909, *P* = 0.06); **compared with HBV-GN, (*χ* ^2^ = 0.400, *P* = 0.527); ***compared with HBV-GN, (*χ* ^2^ = 38.746, *P* < 0.01).

**Table 3:** Expression of AIM2 was negatively correlated with gender, age, and serum e-Ag in HBV-GN.

	*n* (%)	AIM2			*χ* ^2^	*P*
		−	+	++		
Gender, (M)	35 (64.8%)				0.131	0.937
Age (y)					2.598	0.627
≤20	5 (9.3%)	2 (20.0%)	2 (7.7%)	1 (5.6%)		
21–40	29 (53.7%)	4 (40.0%)	16 (61.5%)	9 (50.0%)		
≥41	20 (37.0%)	4 (40.0%)	8 (30.8%)	8 (44.4%)		
e-Ag (+)	33 (70.2%)	6 (60.0%)	16 (69.6%)	11 (78.6%)	0.975	0.614

M: male; e-Ag: HBeAg.

**Table 4:** Expression of AIM2 was negatively correlated with HBV antigen deposition in HBV-GN kidney tissue.

	*n* (%)	AIM2			*χ* ^2^	*P*
		−	+	++		
s-Ag+, c-Ag+	22 (40.7)	5	10	7		
s-Ag+, c-Ag−	24 (44.4)	4	12	8		
s-Ag−, c-Ag+	6 (11.1)	1	4	1		
s-Ag−, c-Ag−	2 (3.7)	0	0	2		

Total	54	10	26	18	5.259	0.511

s-Ag, HBsAg; c-Ag, HBcAg.

**Table 5:** Correlation of AIM2, caspase-1, and IL-1*β* expression in HBV-GN.

AIM2	Caspase-1				IL-1*β*			
	−	+	++	+++	−	+	++	+++
−	7	2	1	0	11	19	4	0
+	4	19	3	0	4	16	7	0
++	2	9	6	1	3	6	8	1

AIM2 was positively correlated with caspase-1 (*r*_*s*_ = 0.444, *P* < 0.01); AIM2 was positively correlated with IL-1*β* (*r*_*s*_ = 0.379, *P* < 0.01); caspase-1 was positively correlated with IL-1*β* (*r*_*s*_ = 0.515, *P* < 0.01).

## Abbreviations

HBV-GN: Hepatitis B virus-associated glomerulonephritis
HAV: Hepatitis A virus
HBV: Hepatitis B virus
HCV: Hepatitis C virus
HDV: Hepatitis D virus
HEV: Hepatitis E virus
HIV: Human immunodeficiency virus
HBsAg: Hepatitis B surface antigen
HBeAg: Hepatitis B e antigen
HBcAg: Hepatitis B core antigen
AIM2: Absent in melanoma 2
IL-1*β*: Interleukin-1b
IL-18: Interleukin-18
HIN-200: Hematopoietic IFN-inducible nuclear protein containing a 200-amino-acid repeat
PYD: Pyrin domain
SPSS: Statistical Package for the Social Sciences
ASC: Apoptosis-associated speck-like protein containing a CARD
CN: Chronic nephritis
CGN: Chronic glomerulonephritis
HGM: Human glomerular mesangial cell line
pro-IL1*β*: pro-Interleukin-1b
dsDNA: Double-stranded DNA
IFN: Interferon
IFI16: Gamma-interferon-inducible protein 16
siRNA: Small interfering RNA
K/DOQI: Kidney Disease Outcome Quality Initiative
NKF: National Kidney Foundation
PBS: Phosphate buffered saline
DAB: Diaminobenzidine
ATCC: American Type Culture Collection
DMEM: Dulbecco's modified Eagle's medium
qRT-PCR: Quantitative reverse transcription polymerase chain reaction
RIPA: Radioimmunoprecipitation assay
BSA: Bovine serum albumin
PVDF: Polyvinylidene fluoride
TBST: Tris-buffered saline with Tween 20
HRP: Horseradish peroxidase
ELISA: Enzyme-linked immunosorbent assay.
